# Using MRI to differentiate upper-lateral intracavitary pregnancy and interstitial pregnancy for the patients with pregnancies in the uterotubal junction during the first trimester

**DOI:** 10.1007/s00330-022-08786-4

**Published:** 2022-04-20

**Authors:** Wenjuan Liu, Weili Xie, Hang Zhao, Xufeng Jiao, Enzhao Sun, Shan Jiang, Ning Zheng, Zhenchang Wang

**Affiliations:** 1 Department of Radiology, Beijing Friendship Hospital, Capital Medical University, Ninety-five Yong’an Road, Xicheng District, Beijing, 100050 China; 2 School of Clinical Medicine, Jining Medical University, Forty-five South Jianshe Road, Jining, 272013 Shandong China; 3 Department of Radiology, Jining No. 1 People’s Hospital, Six Jiankang Road, Jining, 272000 Shandong China

**Keywords:** Ectopic pregnancy, Angular pregnancy, Interstitial pregnancy, Magnetic resonance imaging, First trimester

## Abstract

**Objectives:**

To retrospectively evaluate the diagnostic value of MRI for the uterotubal junctional pregnancies during the first trimester.

**Methods:**

This retrospective study involved 59 patients (January 2016 to July 2021) with a preoperative imaging diagnosis of uterotubal junctional pregnancy. Using operative and pathological reports as the reference standard, we identified 22 patients with upper-lateral intracavitary (angular) pregnancy and 37 patients with interstitial pregnancy. Two senior radiologists, blinded to the patients’ information, reviewed the MRI images and determined each MRI feature based on the original interpretation criteria. Any disagreement was resolved by discussion to achieve a consensus. The sensitivity and specificity of each MRI feature were calculated according to the reference standard.

**Results:**

The endometrial thickness in the upper-lateral intracavitary pregnancy group was larger than in the interstitial group (*p* = 0.001). The cutoff value of the endometrial thickness was 11.5 mm with a sensitivity, specificity, and area under the curve that were 77.3%, 64.9%, and 0.743, respectively. Two key features to diagnose upper-lateral intracavitary pregnancy were “medial free edge” and “medial free edge plus above-cutoff endometrial thickness.” The sensitivity and specificity of the medial free edge were 100% and 94.9%, respectively. The sensitivity and specificity of the medial free edge plus above-cutoff endometrial thickness were 77.3% and 100%, respectively. The key feature to diagnose interstitial pregnancy was an “intact lateral junctional zone,” of which the sensitivity and specificity were 94.6% and 100%, respectively.

**Conclusions:**

MRI can be used to differentiate the upper-lateral intracavitary pregnancy and interstitial pregnancy during the first trimester.

**Key Points:**

• *We demonstrated MRI diagnostic criteria for the interstitial pregnancy and upper-lateral intracavitary pregnancy.*

• *MRI might be used to identify the complex interstitial pregnancies, those with a gestational sac protruding into the uterine cavity.*

## Introduction

Upper-lateral intracavitary pregnancy [1], previously named angular pregnancy [2–4], is a type of potentially viable, eccentric, intracavitary pregnancy located in the cornua of the normal uterus [5, 6]. While deliberate termination of upper-lateral intracavitary pregnancy could avoid the risk of uterine rupture, expectant management is an alternative [4, 7]. If managed appropriately, the live birth rate with upper-lateral intracavitary pregnancy is as high as 80% of cases [3, 4]. It is challenging to differentiate upper-lateral intracavitary pregnancy from other ectopic pregnancies in the uterotubal junction, among which interstitial pregnancy is the most common [5, 6]. Interstitial pregnancy represents 2 to 4% of ectopic pregnancies, but the maternal mortality rate is seven times higher than with other types of ectopic pregnancies, largely owing to massive hemorrhage [8, 9]. It is of great importance to make a precise diagnosis of upper-lateral intracavitary pregnancy and interstitial pregnancy during the first trimester, since a misdiagnosis of these two conditions may result in either the termination of a viable pregnancy [6, 7] or life-threatening obstetric emergencies [10–12].

First trimester ultrasonography is a fundamental imaging approach for assessing patients with suspected ectopic pregnancies. However, the precise ultrasonographic diagnosis may not be achieved during the early stage of pregnancy as this imaging approach is highly dependent on the operator’s experience [1]. In addition, the unusual implantation sites of upper-lateral intracavitary and interstitial pregnancies, which may also coexist with heavy hemorrhage, bowel gas, and ovarian masses, may interfere with the diagnostic accuracy of this imaging modality [13–15]. With the excellent intra- and inter-observer agreements [16, 17] as well as the superior soft tissue contrast, magnetic resonance imaging (MRI) can identify unusual implantation sites and distinguish fresh blood from other fluid collections in multi-sectional views [14, 16, 18–21]. Owing to the distinct implantation sites and the outcomes of embryo development, the gestational sacs in interstitial pregnancy and upper-lateral intracavitary pregnancy may demonstrate different anatomical and physiological relationships with the surrounding soft tissues and organs, such as the uterine cavity, endometrium, and parauterine blood vessels. Therefore, MRI may have unique advantages for the differential diagnosis of upper-lateral intracavitary pregnancy and interstitial pregnancy, which are easily misdiagnosed and confused with each other, especially once the gestational sac of interstitial pregnancy protrudes into the uterine cavity during the first trimester [1, 7, 10].

Using operative and pathological reports as the reference standard, we established a retrospective cohort of patients preoperatively diagnosed with uterotubal junctional pregnancies. We hypothesized that upper-lateral intracavitary pregnancy and interstitial pregnancy can be precisely differentiated by MRI.

## Materials and methods

### Patients

**Fig. 1:**
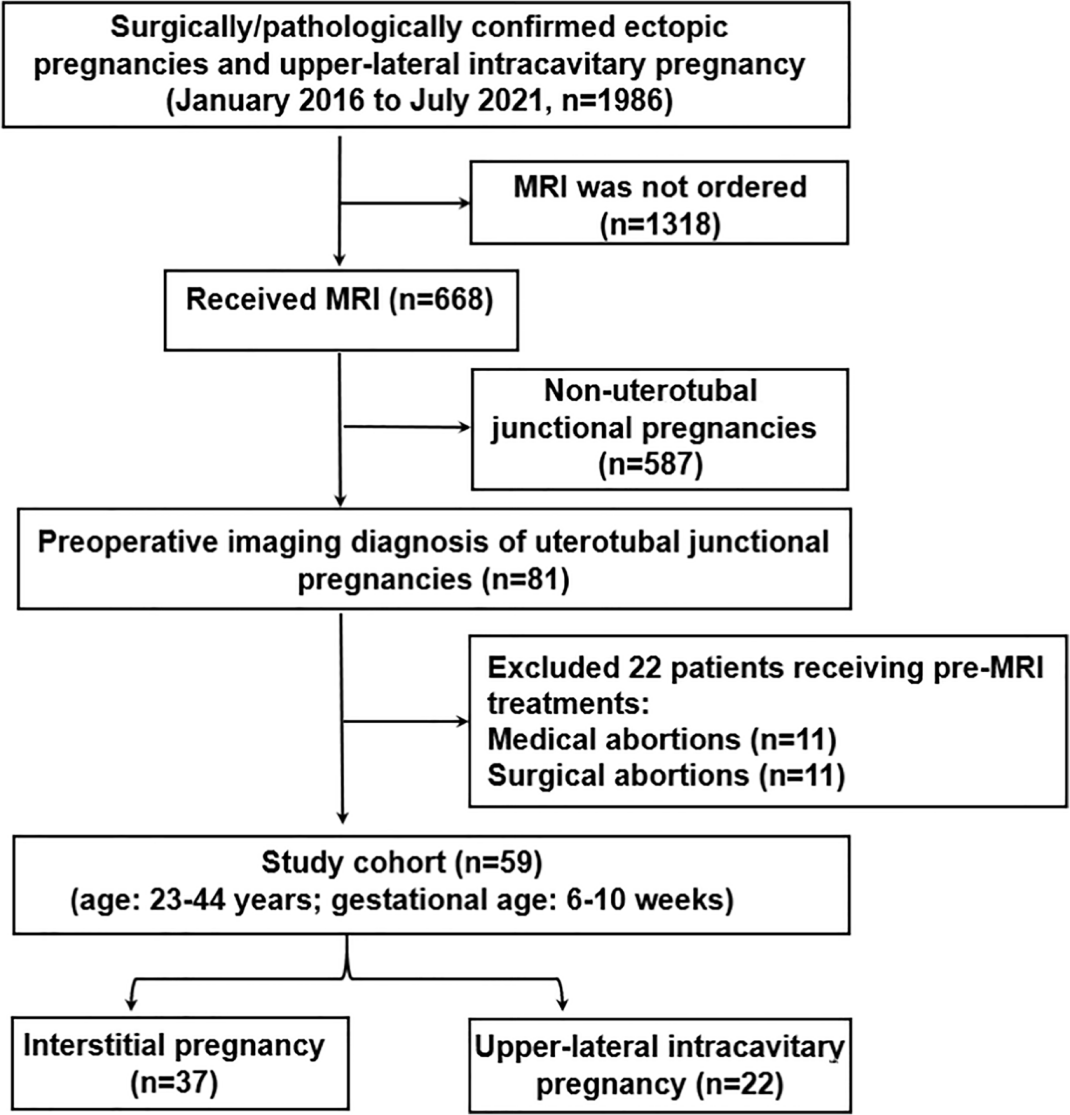
Flow chart of the study design

Our hospital’s institutional review board approved this study, and the need to obtain consent for this retrospective analysis was waived by the board. We consecutively reviewed the patients’ medical records in our institution (January 2016 to July 2021). As shown in Fig. 1, 1986 patients were confirmed with ectopic pregnancies or upper-lateral intracavitary pregnancy according to operative and pathological reports. We excluded 1318 cases with precise ultrasonographic diagnosis so that the MRI was not ordered. For patients with equivocal ultrasonographic view and/or complications (*n* = 668), written informed consent for MRI was obtained. We excluded 587 patients whose MRI diagnosis was non-uterotubal junctional pregnancy. Among the remaining 81 patients with a diagnosis of uterotubal junctional pregnancy, we excluded 22 patients receiving pre-MRI medical (*n* = 11) or surgical (*n* = 11) abortion, as these treatments might affect the gestational sac and endometrial thickness. The final study cohort comprised 59 patients (age: 23–44 years; gestational age: 6–10 weeks) with a preoperative diagnosis of either upper-lateral intracavitary pregnancy (*n* = 22) or interstitial pregnancy (*n* = 37).

### Reference standard

The presence of a gestational sac was confirmed by the microscopic view of chorionic villi and/or an extravillous trophoblast. Laparoscopy was used to make the differential diagnosis of upper-lateral intracavitary pregnancy and interstitial pregnancy. Upper-lateral intracavitary pregnancy was confirmed by directly observing a laterally distended uterus accompanied by lateral displacement of the round ligament. Interstitial pregnancy was diagnosed laparoscopically by confirming that the round ligament was located medial to the swelling at the uterotubal junction.

### MRI protocol

While 1.5-T MRI is widely used for fetal imaging examination [22], 3-T MRI has been shown be a safe approach with superior signal-to-noise ratio and spatial resolution [23]. In the current study, MRI was performed on an Ingenia 3-T (Philips Healthcare) using a 16-channel anterior phased array coil plus 12-channel built-in table coils. Axial, coronal, and sagittal images were collected. Each plane has 24 slices. MRI sequences comprised axial T1-weighted turbo spin echo (TSE) images (TR/TE: 400–600/14 ms; FOV, 240 mm × 351 mm; voxel size, 0.9 mm × 1.0 mm × 5.0 mm; gap, 1 mm; reconstruction in-plane resolution 0.49 mm^2^; NSA 1.6; acquisition time, 1 m 55 s), axial T2-weighted spectral presaturation with inversion recovery (SPIR) MultiVane images (TR/TE: 2500–5000/100 ms; FOV, 300 mm × 300 mm; voxel size, 0.9 mm × 0.9 mm × 5.0 mm; gap, 1 mm; reconstruction in-plane resolution 0.57 mm^2^; TSE factor 27; NSA 1; acquisition time, 3 m 12 s), sagittal T2-weighted SPIR MultiVane images (TR/TE: 3000–5000/100 ms; FOV, 250 mm × 250 mm; voxel size, 0.84 mm × 0.84 mm × 4 mm; gap, 1 mm; reconstruction in-plane resolution 0.63 mm^2^; TSE factor 28; NSA 1; acquisition time, 3 m 21 s), and coronal T2-weighted TSE images with SENSE (TR/TE: 1700–5000/130 ms; FOV, 260 mm × 350 mm; voxel size, 0.8 mm × 1.0 mm × 5.0 mm; gap, 1 mm; reconstruction in-plane resolution 0.68 mm^2^ ;TSE factor 22; NSA 2; SENSE, 2.5; acquisition time, 1 m 38 s).

### MRI image analysis

All images were reviewed independently by two senior radiologists with 20 and 10 years of experience respectively. Both MRI reviewers were blinded to the patient’s medical history, surgical findings, and pathological reports. Any disagreement was resolved by discussion to achieve a consensus.

**Fig. 2:**
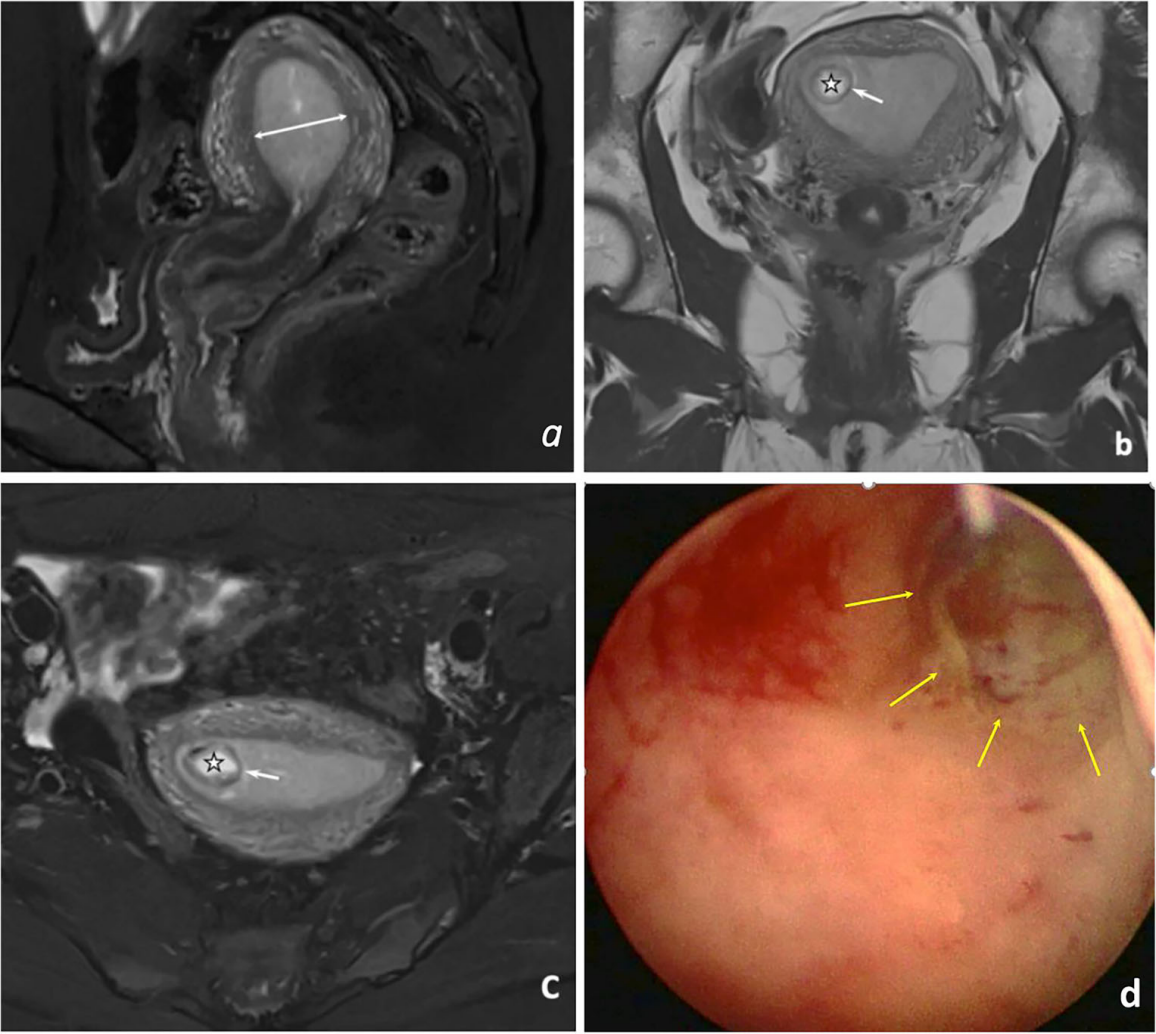
MRI and hysteroscopy in a 24-year-old patient with upper-lateral intracavitary pregnancy. **a** Sagittal fat-suppressed T2WI showing an endometrial thickness (double-ended arrow) of 22.0 mm. **b** Coronal T2WI and (**c**) axial fat-suppressed T2WI showing the gestational sac (star) and medial free edge (arrow). **d** Hysteroscopic view showing the intrauterine exposed portion of the gestational sac forming a free edge (yellow arrows)

**Fig. 3:**
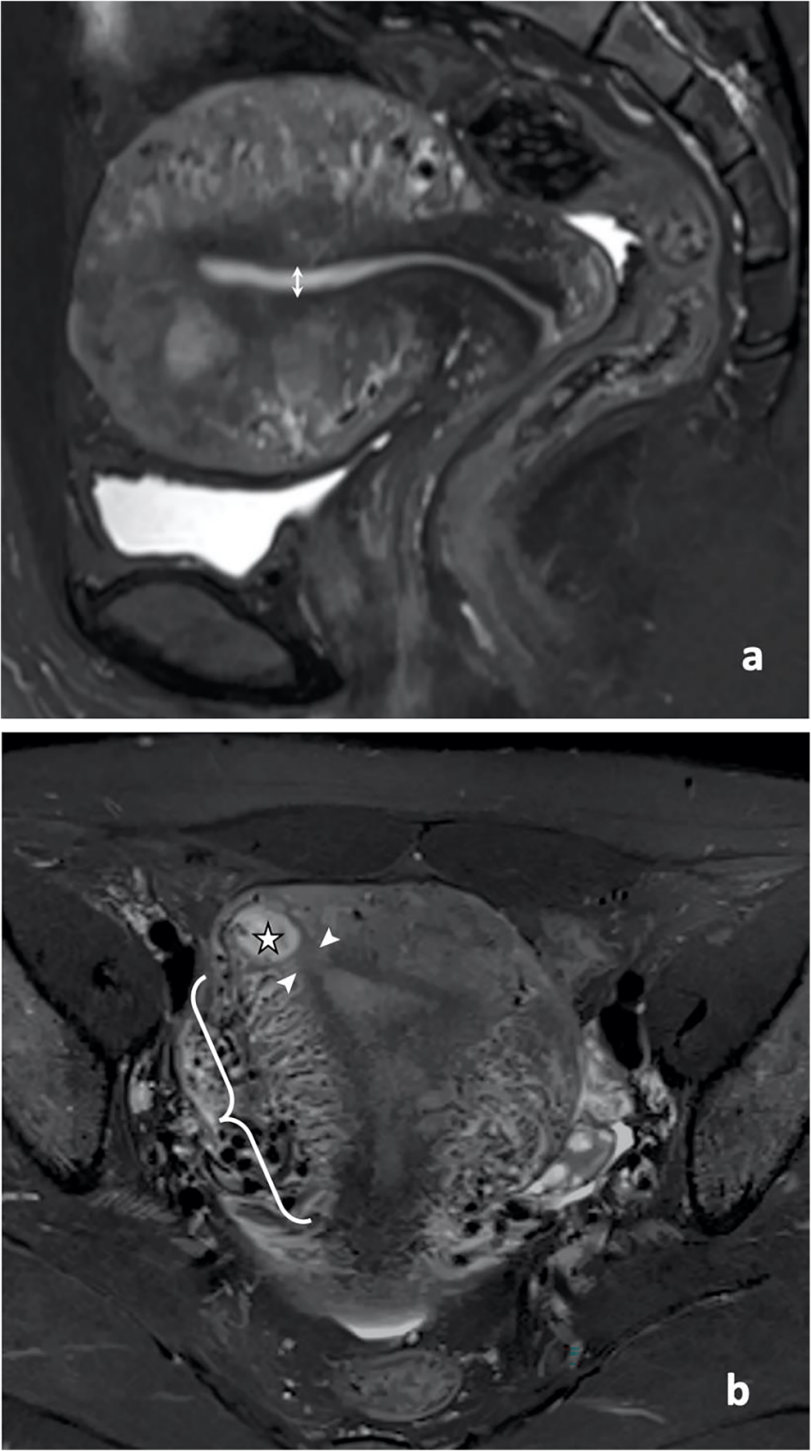
Fat-suppressed, T2-weighted MRI in a 34-year-old patient with interstitial pregnancy. **a** Sagittal image showing an endometrial thickness (double-ended arrow) of 3.6 mm. **b** Coronal image showing the gestational sac (star), intact lateral junctional zone (arrowheads), and ipsilateral flow-void (curly bracket)

**Fig. 4:**
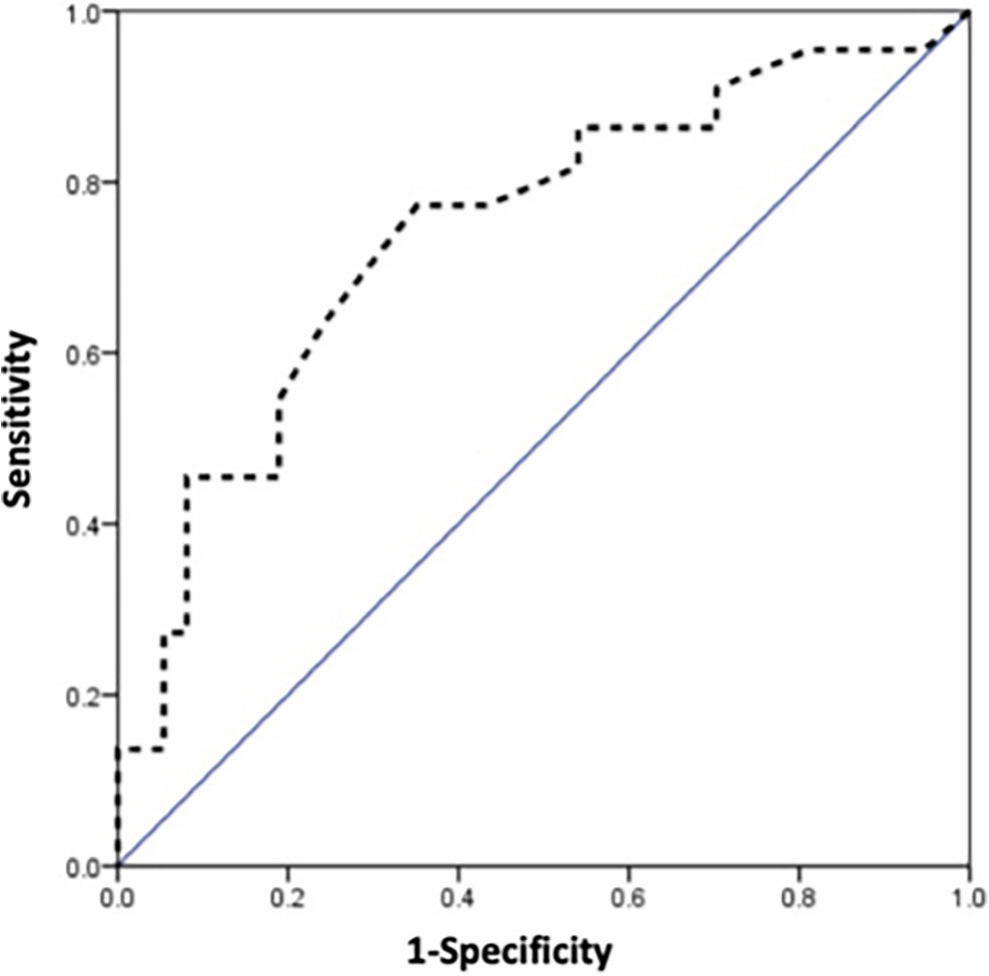
Receiver operating characteristic curve of endometrial thickness. The cutoff value of the endometrial thickness is 11.5 mm with a sensitivity, specificity, and area under the curve of 77.3%, 64.9%, and 0.743, respectively

The MRI review focused on the anatomical relationship between the gestational sac and uterine cavity as well as physiological changes in endometrial thickness and parauterine blood flow. Endometrial thickness was measured in fat-suppressed, T2-weighted sagittal images (Figs. 2a and 3a). The cutoff value for endometrial thickness was determined using a receiver operating characteristic curve (Fig. 4). For upper-lateral intracavitary pregnancy, three MRI findings were examined: medial free edge, above-cutoff endometrial thickness, and the combination of these two signs. For interstitial pregnancy, we investigated an intact lateral junctional zone, ipsilateral parauterine flow-void, and the combination of ipsilateral flow-void and below-cutoff endometrial thickness. A medial free edge was defined as the portion of the gestational sac wall directly exposed to the uterine cavity (Fig. 2b and c). An intact lateral junctional zone was defined as an uninterrupted zone located lateral to the uterus and between the uterine cavity and the external tangent gestational sac (Fig. 3b) [13, 18, 19]. A flow-void sign was characterized as parauterine multiple dot-like or tubular structures with low signal intensity (Fig. 3b) [24].

### Statistical analysis

Data were analyzed using SPSS 20.0 software (IBM Inc.). Numerical data, including the patient’s age, gestational age, ß-hCG concentration, and sagittal endometrial thickness, were assessed for normality distribution and homogeneous variance by the Shapiro–Wilk test and Levene test, respectively. As all the data sets met assumptions of homogeneity of variance and normality, we used the Student *t* test to compare the mean of each parameter between the upper-lateral intracavitary pregnancy group and interstitial pregnancy group. Receiver operating characteristic curve was constructed to determine the cutoff value for the endometrial thickness measured in sagittal images. Fisher’s exact test was used to analyze categorical variables including the presence or absence of abdominal pain, vaginal bleeding, and intrauterine hemorrhage. A *p* value less than 0.05 was considered statistically significant. For each MRI finding, we analyzed the sensitivity, specificity, positive predictive value (PPV), negative predictive value (NPV), positive likelihood ratio (LR+), negative likelihood ratio (LR−), and diagnostic accuracy (DA).

## Results

### Patient’s demographics and clinical characteristics

As shown in Table 1, the ß-hCG concentration differed significantly between the upper-lateral intracavitary pregnancy group and the interstitial pregnancy group (*p*=0.001). We found no difference regarding age, gestational age, the presence of abdominal pain, and/or vaginal bleeding.

**Table 1:** Patient’s demographics and clinical characteristics

	Upper-lateral intracavitary pregnancy (*n*=22)	Interstitial pregnancy (*n* = 37)	*p* value
Age (year)	31.5 ± 4.8 (29.3–33.6)^a^	34.8 ± 5.0 (32.1–36.0)^a^	0.250^b^
Gestational age (week)	8.1 ± 1.1 (7.6–8.6)^a^	7.7 ± 1.0 (7.3–8.1)^a^	0.172 ^b^
Abdominal pain	4/22 (18.2%)	13/37 (35.1%)	0.237^c^
β-hCG (mIU/mL)	63,664 ± 60,627 (36,784–90,544)^a^	25,704 ± 27,378 (13,951–31,845)^a^	0.001 ^b^
Vaginal bleeding	10/22 (45.5%)	19/37 (51.4%)	0.789 ^c^
Endometrial thickness (mm)	15.0 ± 6.6 (12.0–17.9)^a^	9.4 ± 6.6 (7.2–12.3)^a^	0.001 ^b^
Intrauterine hemorrhage	6/22 (27.3%)	4/37 (10.8%)	0.152 ^c^

^a^95% confidence interval (CI)

^b^Student’s *t* test

^c^Fisher’s exact test

We measured the endometrial thickness in T2-weighted sagittal images (Figs. 2a and 3a) and found that the upper-lateral intracavitary pregnancy group had significantly higher thickness than the interstitial pregnancy group (*p* = 0.001). The cutoff value for endometrial thickness was 11.5 mm, with a sensitivity, specificity, and area under the curve of 77.3%, 64.9%, and 0.743, respectively (Fig. 4).

### Upper-lateral intracavitary pregnancy

**Fig. 5:**
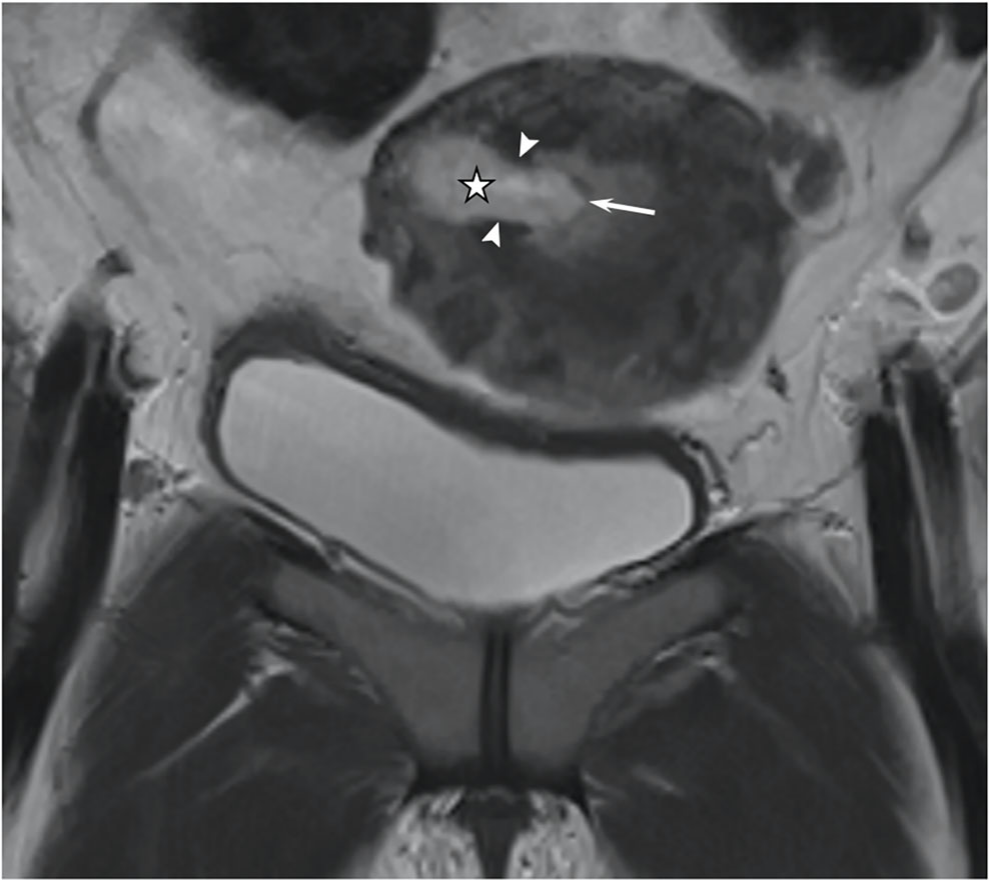
MRI in a 30-year-old patient with interstitial pregnancy. Coronal T2-weighted image showing the gestational sac (star), medial free edge (arrow), and interrupted lateral junctional zone (arrowheads)

As a single MRI feature to diagnose upper-lateral intracavitary pregnancy, the sensitivity, specificity, PPV, NPV, and DA for a medial free edge (Fig. 2b and c) were 100%, 94.9%, 91.7%, 100%, and 95.2%, respectively (Table 2). A medial free edge formed because of the partial, intracavitary exposure of an eccentrically implanted gestational sac (Fig. 2d). The sensitivity and specificity of the above-cutoff endometrial thickness were 77.3% and 66.7%, respectively. Two patients with interstitial pregnancy were false-positive for the sign of medial free edge because a portion of the gestational sac was directly exposed to the uterine cavity (Fig. 5, representative image). The combination of above-cutoff endometrial thickness and medial free edge dramatically increased both specificity and PPV to 100% (Table 2), as both false-positive patients with an endometrium thinner than 11.5 mm were excluded.

**Table 2:** Diagnostic value of each MRI feature for the differential diagnosis of upper-lateral intracavitary pregnancy

	Upper-lateral intracavitary pregnancy		Sn (%)	Sp (%)	PPV (%)	NPV (%)	LR+	LR−	DA (%)
	Yes (*n* = 22)	No (*n* = 37)							
Medial free edge	22	2	100	94.9	91.7	100	19.5	0	95.2
Endometrium ≥ 11.5 mm	17	13	77.3	66.7	56.7	83.9	2.32	0.34	69.4
Medial free edge + endometrium ≥ 11.5 mm	17	0	77.3	100	100	88.6	+∞	0.23	90.3

*Sn*, sensitivity; *Sp*, specificity; *PPV*, positive predictive value; *NPV*, negative predictive value; *LR+*, positive likelihood ratio; *LR−*, Negative likelihood ratio; *DA*, diagnostic accuracy

### Interstitial pregnancy

The single key feature to diagnose interstitial pregnancy was an intact lateral junctional zone (Fig. 3b). The sensitivity, specificity, PPV, NPV, and DA of this feature were 94.6%, 100%, 100%, 92.3%, and 96.7%, respectively (Table 3). The false-negative rate for this sign was 5.4% (2/37) as two patients showed an interruption of the junctional zone (Fig. 5, representative image). As a single feature, the sensitivity and specificity of enhanced ipsilateral flow-void (Fig. 3b) were 64.9% and 81.8%, respectively, which were much lower than the values for an intact lateral junctional zone (Table 3). The combination of enhanced ipsilateral flow-void and below-cutoff endometrial thickness increased the specificity to 90.9%, but the sensitivity decreased to 43.2% (Table 3).

**Table 3:** Diagnostic value of each MRI feature for the differential diagnosis of interstitial pregnancy

	Interstitial	pregnancy	Sn (%)	Sp (%)	PPV (%)	NPV (%)	LR+	LR−	DA (%)
	Yes (*n* = 37)	No (*n* = 22)							
Intact lateral junctional zone	35	0	94.6	100	100	92.3	+∞	0.054	96.7
Enhanced ipsilateral flow-void	24	4	64.9	81.8	85.7	58.1	3.57	0.43	71.2
Enhanced ipsilateral flow-void + endometrium < 11.5 mm	16	2	43.2	90.9	88.9	48.8	4.76	0.62	61.0

*Sn*, sensitivity; *Sp*, specificity; *PPV*, positive predictive value; *NPV*, negative predictive value; *LR+*, positive likelihood ratio; *LR−*, negative likelihood ratio; *DA*, diagnostic accuracy

## Discussion

In the present study, we used operative and pathological reports as the reference standard to establish a retrospective cohort of patients with a preoperative imaging diagnosis of uterotubal junctional pregnancy. We identified three key MRI features, namely, medial free edge, medial free edge plus above-cutoff endometrial thickness, and intact lateral junctional zone, which could be used to make the differential diagnosis of upper-lateral intracavitary pregnancy and interstitial pregnancy.

The medial free edge was a novel MRI feature to diagnose upper-lateral intracavitary pregnancy. Hysteroscopy revealed that a medial free edge formed because of partial exposure of the gestational sac to the uterine cavity, as the eccentrically located gestational sac was not completely surrounded by myometrial tissue. Similar to the medial free edge we demonstrated, Bradly et al [25] and Grant et al [7] identified “double sac” and “surrounding endometrium” by ultrasonography. The MRI and hysteroscopic results in our study, together with the previous ultrasonographic findings [7, 25], indicate that the medial free edge is a highly sensitive feature for the diagnosis of upper-lateral intracavitary pregnancy.

Endometrial thickness is an independent risk factor that has been used to predict the occurrence of ectopic pregnancy [26–29]. Similar to findings in previous publications [26–28, 30], the endometrial thickness in the upper-lateral intracavitary pregnancy group was significantly higher than in the interstitial pregnancy group, in our study. After combining the above-cutoff (11.5 mm) endometrial thickness and medial free edge, the specificity was increased to 100% since the two patients who were false-positive for the medial free edge sign had an endometrial thickness that was less than 11.5 mm. The introduction of endometrial thickness is specifically useful to distinguish the upper-lateral intracavitary pregnancy and the interstitial pregnancies with a gestational sac protruding into the uterine cavity [1]. Given that the endometrial thickness is proportional to the pregnancy viability [27, 31] and a medial free edge indicates the eccentric, upper-lateral intracavitary implantation of a gestational sac, the extremely high specificity of the combination of these two signs suggests the high concordance between MRI features and the biological characteristics of upper-lateral intracavitary pregnancy, which is a potentially viable pregnancy implanting in the uterine cornua.

An intact lateral junctional zone was the key feature for diagnosing interstitial pregnancy. While the sign of intact lateral junctional zone has been previously reported [13, 18, 19], we further confirmed this sign not only by introducing a solid reference standard and using larger sample size, but also by emphasizing the anatomical location that is between the uterine cavity and the external tangent gestational sac. The false-negative rate for this sign was 5.4% (2/37) since the two false-negative patients showed gestational sacs that protruded into the uterine cavity, resulting in an interrupted junctional zone. Not surprisingly, the same patients were also false-positive for the sign of medial free edge. We assumed that the gestational sac in both patients may have initially implanted in a proximal portion of the interstitium; however, more evidence is yet to be demonstrated.

The presence of flow-void in MRI suggests enhanced blood flow through dilated blood vessels [24, 32]. During pregnancy, parauterine blood vessels undergo drastic remodeling to facilitate the dramatic increase in uteroplacental blood flow that is required for normal pregnancy outcomes [33]. Thus, the sign of enhanced ipsilateral flow-void in the patients with interstitial pregnancy might be owing to the ectopically implanted gestational sac that facilitates the proliferation of ipsilateral blood vessels. After combining ipsilateral flow-void and below-cutoff endometrial thickness, the specificity for interstitial pregnancy reached 90.9%. Adding the below-cutoff endometrial thickness excluded two out of four upper-lateral intracavitary pregnancy patients whose endometrium was thicker than 11.5 mm but who were false-positive for the sign of enhanced ipsilateral flow-void. Therefore, the combination of enhanced ipsilateral flow-void and below-cutoff endometrial thickness could be used as a supplementary feature to diagnose interstitial pregnancy.

There are two major limitations to our study. First, this was a retrospective study with a starting population comprising patients with a confirmed diagnosis of ectopic pregnancy or upper-lateral intracavitary pregnancy. Therefore, the patients with uterotubal masses that were not ectopic pregnancies were excluded from our study. Although the presence of patients with uterotubal junctional masses might interfere with the diagnostic value of the key features demonstrated in this study, the differential diagnosis of suspected uterotubal junctional masses is not made solely using MRI findings. Clinical manifestations, ß-hCG concentrations, and ultrasonographic findings all provide valuable information, based on which the diagnostic accuracy of MRI would be largely maintained. The second limitation of our study is the potentially overstated accuracy of the MRI-designated uterine cornua and uterotubal junction. The uterine cornua lacks an absolute anatomical boundary, while the uterotubal junction can only be defined by pathological examination [5, 6]. Consequently, an imaging diagnosis of uterotubal junctional pregnancy may somehow be equivocal. A well-designed prospective study may overcome these limitations, while a consensus is also necessary regarding the imaging-based definition of uterine cornua and uterotubal junction.

In conclusion, MRI is a valuable alternative to ultrasonography for diagnosing upper-lateral intracavitary and interstitial pregnancies, especially once the gestational sac of interstitial pregnancy protrudes into the uterine cavity. The evidence we provided will contribute to both achieving the precise diagnosis of these two entities and optimizing therapeutic strategies, particularly expeditious decision-making between deliberate termination and expectant management.

## Abbreviations

DA: Diagnostic accuracy
LR−: Negative likelihood ratio
LR+: Positive likelihood ratio
MRI: Magnetic resonance imaging
NPV: Negative predictive value
PPV: Positive predictive value
Sn: Sensitivity
Sp: Specificity

## Declarations

### Guarantor

The scientific guarantor of this publication is Zhenchang Wang, Professor of Medicine, Department of Radiology, Capital Medical University affiliated Beijing Friendship Hospital.

### Statistics and biometry

No complex statistical methods were necessary for this paper.

### Methodology

• Retrospective
